# Cardio-Ankle Vascular Index Reflects Impaired Exercise Capacity and Predicts Adverse Prognosis in Patients With Heart Failure

**DOI:** 10.3389/fcvm.2021.631807

**Published:** 2021-03-29

**Authors:** Koichiro Watanabe, Akiomi Yoshihisa, Yu Sato, Yu Hotsuki, Fumiya Anzai, Yasuhiro Ichijo, Yusuke Kimishima, Tetsuro Yokokawa, Tomofumi Misaka, Takamasa Sato, Takashi Kaneshiro, Masayoshi Oikawa, Atsushi Kobayashi, Yasuchika Takeishi

**Affiliations:** ^1^Department of Cardiovascular Medicine, Fukushima Medical University, Fukushima, Japan; ^2^Department of Advanced Cardiac Therapeutics, Fukushima Medical University, Fukushima, Japan

**Keywords:** cardio-ankle vascular index, arterial stiffness, cardiopulmonary exercise testing, heart failure, prognosis

## Abstract

**Aims:** We aimed to assess the associations of CAVI with exercise capacity in heart failure (HF) patients. In addition, we further examined their prognosis.

**Methods:** We collected the clinical data of 223 patients who had been hospitalized for decompensated HF and had undergone both CAVI and cardiopulmonary exercise testing.

**Results:** For the prediction of an impaired peak oxygen uptake (VO_2_) of < 14 mL/kg/min, receiver-operating characteristic curve demonstrated that the cutoff value of CAVI was 8.9. In the multivariate logistic regression analysis for predicting impaired peak VO_2_, high CAVI was found to be an independent factor (odds ratio 2.343, *P* = 0.045). We divided these patients based on CAVI: the low-CAVI group (CAVI < 8.9, *n* = 145) and the high-CAVI group (CAVI ≥ 8.9, *n* = 78). Patient characteristics and post-discharge cardiac events were compared between the two groups. The high-CAVI group was older (69.0 vs. 58.0 years old, *P* < 0.001) and had lower body mass index (23.0 vs. 24.1 kg/m^2^, *P* = 0.013). During the post-discharge follow-up period of median 1,623 days, 58 cardiac events occurred. The Kaplan–Meier analysis demonstrated that the cardiac event rate was higher in the high-CAVI group than in the low-CAVI group (log–rank *P* = 0.004). The multivariate Cox proportional hazard analysis revealed that high CAVI was an independent predictor of cardiac events (hazard ratio 1.845, *P* = 0.035).

**Conclusion:** High CAVI is independently associated with impaired exercise capacity and a high cardiac event rate in HF patients.

## Introduction

Impaired exercise capacity is an independent predictor of poor prognosis in patients with heart failure (HF) (1–3). Cardiopulmonary exercise testing (CPX) is the widely accepted gold-standard approach to assess exercise capacity (4). However, compared with other exercise tests (6-min walk test, electrocardiography stress testing), CPX is more time-consuming, more expensive, and needs specialized equipment and personnel (4). Vascular dysfunction (e.g., arterial stiffness, endothelial dysfunction) in HF may contribute to altered ventricular-arterial coupling (4), and might be associated with impaired exercise capacity (4, 5). The cardio-ankle vascular index (CAVI) is a measure of arterial stiffness, and is useful to evaluate atherosclerosis, and moreover to predict the prognosis in patients who have multiple risk factors of cardiovascular diseases (6–9). High CAVI is an independent predictor of cardiovascular events including cardiovascular death, nonfatal myocardial infarction, or nonfatal ischemic stroke in patients with acute coronary syndrome (7).

However, the clinical implication of CAVI in patients with HF is yet unclear, especially in terms of assessing exercise capacity and prognosis. Therefore, we aimed to assess the associations of CAVI with exercise capacity in HF patients. In addition, we further examined their prognosis.

## Methods

### Subjects and Protocol

**Figure 1:**
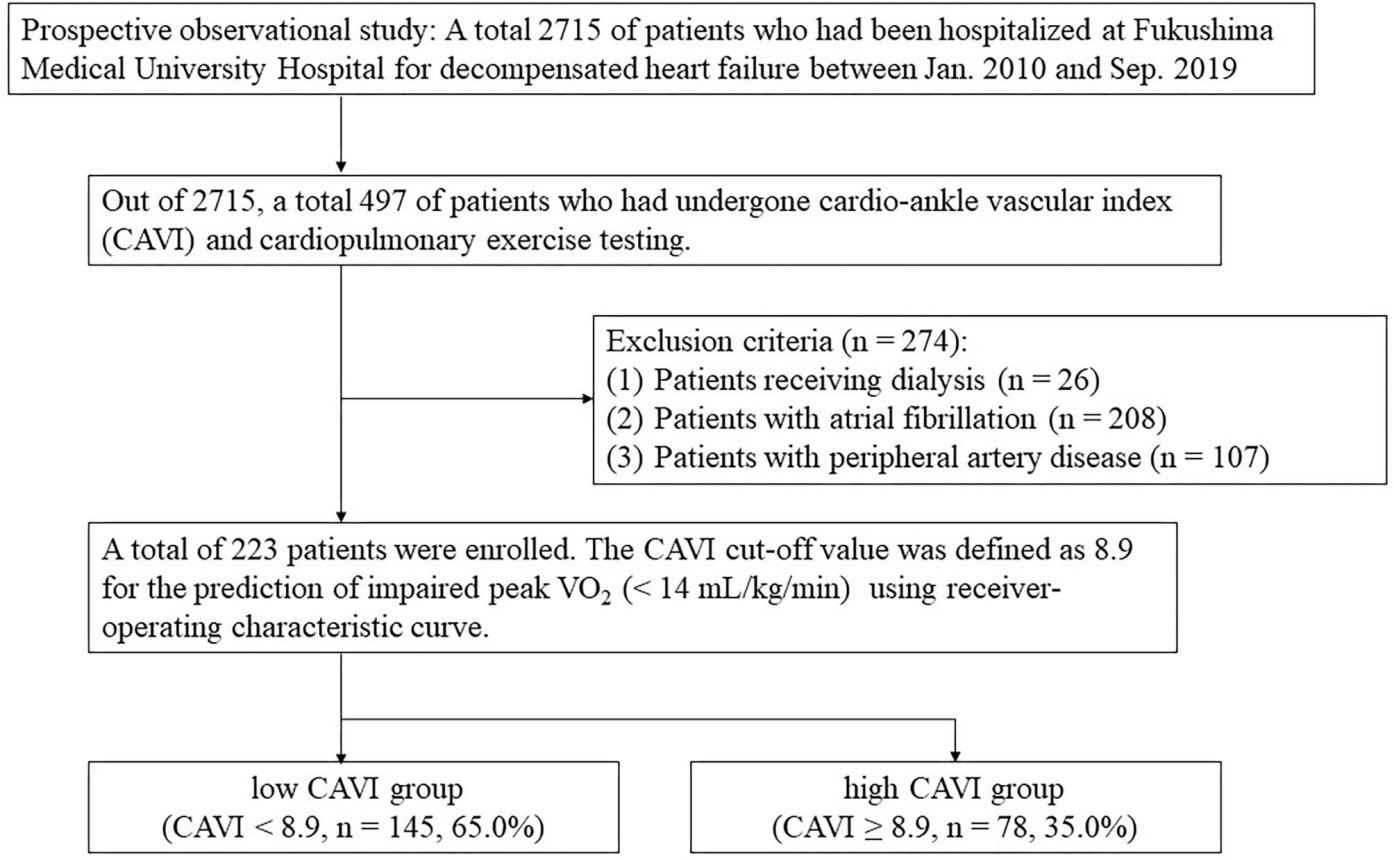
Patient flow chart.

**Figure 2:**
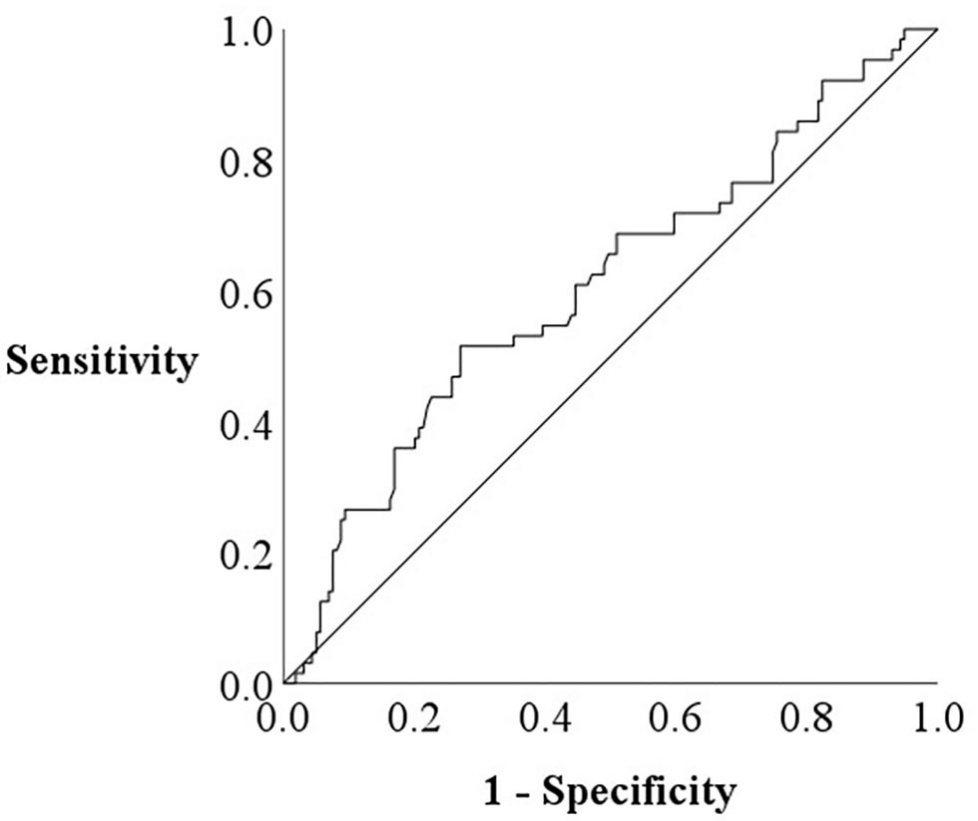
Receiver-operating characteristic curve for the prediction of impaired peak VO_2_ by CAVI.

The patient flow chart is presented in Figure 1. This was a prospective observational study of patients who (1) had been both hospitalized at Fukushima Medical University Hospital for decompensated HF and discharged alive between January 2010 and September 2019; and (2) out of the 2,715 HF patients, a total of 497 patients had undergone both CAVI measurement and CPX testing before discharge in a stable condition. Patients with decompensated HF were identified by the current guidelines (1, 2). Patients with obvious history of peripheral artery disease, those with atrial fibrillation and/or those who were receiving maintenance dialysis throughout the study period were excluded (*n* = 274). We excluded patients with concurrent peripheral artery disease and atrial fibrillation because it is difficult to accurately measure CAVI in such patients (patients receiving dialysis *n* = 26 and/or patients with atrial fibrillation *n* = 208 and/or patients with peripheral artery disease *n* = 107) (7). Peripheral artery disease was defined as in previous studies (10, 11). Other co-morbidities were also defined in accordance with our previous studies (10, 11). We defined reduced ejection fraction (EF) as left ventricular EF (LVEF) < 40%, mid-range EF as 40% ≤ LVEF < 50% and preserved EF ≥ 50% (1–3). Finally, a total of 223 patients were enrolled. For the prediction of impaired peak VO_2_, defined as < 14 mL/kg/min (12), receiver-operating characteristic (ROC) curve demonstrated that the cut-off value of CAVI was 8.9 (Figure 2, area under curve 0.67, 95% confidence interval, 0.52–0.69, *P* < 0.05). Next, these patients were divided into two groups based on this cut-off value: the low-CAVI group (CAVI < 8.9, *n* = 145, 65.0%) and the high-CAVI group (CAVI ≥ 8.9, *n* = 78, 35.0%). Patient characteristics and post-discharge prognosis were compared between the two groups. The patient characteristics included demographic data at discharge, as well as laboratory data and echocardiographic data, which were obtained within one week prior to discharge when the patient was in a stable condition. We compared post-discharge cardiac events, ischemic events and all-cause mortality.

These patients were followed up until March 2020 for cardiac events as composites of cardiac death or unplanned re-hospitalization for HF treatment, ischemic coronary events and all-cause mortality. For patients that experienced two or more events, only the first event was included in the analysis. Since these patients visited patient's referring hospital monthly or bi-monthly, we were able to follow up on all patients. Status and dates of death were obtained from the patient's medical records. This study conformed to the Declaration of Helsinki (13) and the statement of STROBE (Strengthening the Reporting of Observational studies in Epidemiology) (14). The ethical committee of Fukushima Medical University approved the study protocol. Written informed consent was obtained from all patients.

### The Measurement of CAVI

We measured CAVI automatically by using VaSera VS-1000 (Fukuda Denshi Co., Ltd., Tokyo, Japan) with the patient in the decubitus position before discharge in a stable condition. We attached cuffs bilaterally to the upper arms and ankles of the patient. We placed electrocardiogram electrodes and a microphone on both wrists and on the sternum, respectively. We analyzed the average CAVI values of both sides (6–9).

### Cardiopulmonary Exercise Testing

Patients underwent incremental symptom-limited exercise testing before discharge in a stable condition, using an upright cycle ergometer with a ramp protocol (Strength Ergo 8, Fukuda Denshi Co., Ltd., Tokyo, Japan). Breath-by-breath VO_2_ was measured during exercise using an Aeromonitor AE-300S (Minato Medical Science Co., Ltd., Osaka, Japan). Breath-by-breath oxygen consumption (VO_2_), carbon dioxide production (VCO_2_), and minute ventilation (VE) were measured during exercise using an AE-300S respiratory monitor (Minato Medical Science, Co., Ltd.) (15). Peak VO_2_ was measured as an average of the last 30 s of exercise, and ventilatory response to exercise (slope of the relationship between ventilation and carbon dioxide production, VE/VCO_2_ slope) was calculated as the regression slope relating VE to CO_2_ from the start of exercise until the respiratory compensation point (the time at which ventilation is stimulated by CO_2_ output and end-tidal CO_2_ tension begins to decrease) (15). We calculated the ventilatory anaerobic threshold using the V-slope method.

### Statistical Analysis

Normality was confirmed using the Shapiro-Wilk test in each group. Normally distributed variables are presented as mean ± standard deviation, non-normally distributed variables are presented as median (interquartile range), and categorical variables are expressed as counts and percentages. ROC curves for predicting impaired peak VO_2_ were plotted using EZR version 1.40 (Saitama Medical Center, Jichi Medical University, Saitama, Japan) (16). Non-normally distributed variables were compared using the Mann-Whitney U test, and the Fisher's exact test was used for comparisons of categorical variables. If 20% or more cells had expected count less than five, the one-sided Fisher's exact test was adopted. Logistic regression analysis was performed to assess associations between impaired exercise capacity and CAVI, as well as other variables (e.g., age, sex, blood pressure, heart rate, hypertension, diabetes mellitus, dyslipidemia, coronary artery disease, cerebral vascular disease, chronic kidney disease, anemia, BNP, and LVEF), which are generally thought to be associated with exercise capacity. The occurrence of post-discharge cardiac events, ischemic events and all-cause mortality was compared using the Kaplan-Meier analysis with a log-rank test. We assessed CAVI as a predictor for cardiac events, ischemic coronary events and all-cause mortality using the univariate or multivariate Cox proportional hazard analysis. The threshold for statistical significance was *P* < 0.05. All analyses, except for ROC, were conducted using IBM SPSS Statistics version 26 (IBM, Armonk, NY, USA).

### Data Availability

The data that support the findings of this study are available from the corresponding author upon reasonable request.

## Results

Comparisons of patient characteristics between the low- and high-CAVI groups are shown in Table 1. A total of 78 (35.0%) patients belonged to the high-CAVI group. The high-CAVI group was older and showed lower body mass index. Prevalence of hypertension and chronic kidney disease was significantly higher in the high-CAVI group than in the low-CAVI group. In contrast, sex, blood pressure, heart rate, NYHA functional class, other co-morbidities, and medications did not differ between the two groups. There were no significant differences regarding BNP levels and LVEF between the two groups.

**Table 1:** Baseline patient characteristics.

	**Low CAVI (CAVI < 8.9, *n* = 145)**	**High CAVI (CAVI ≥ 8.9, *n* = 78)**	***P* value**
**CAVI**	7.31 (6.50–8.00)	9.62 (9.36–10.14)	<0.001
**Demographic data**			
Age (years old)	58.0 (46.0–65.0)	69.0 (61.0–74.0)	<0.001
Male sex (*n*, %)	112 (77.2)	66 (84.6)	0.191
Body mass index (kg/m^2^)	24.1 (22.2–28.1)	23.0 (21.4–26.3)	0.013
Systolic blood pressure (mmHg)	122.5 (108.0–143.0)	130.0 (115.0–151.5)	0.094
Diastolic blood pressure (mmHg)	71.5 (61.0–86.0)	77.0 (62.0–91.0)	0.436
Heart rate (/min)	78.0 (65.0–96.0)	73.0 (62.0–89.5)	0.140
NYHA functional class III or IV(*n*, %)	1 (0.7)	1 (1.3)	0.578
Ischemic etiology (*n*, %)	41 (32.5)	29 (44.6)	0.070
Reduced/mid-range/preserved EF (*n*, %)	48 (38.4)/24 (19.2)/53 (42.4)	19 (29.7)/14 (21.9)/31 (48.8)	0.496
**Co-morbidities**			
Hypertension (*n*, %)	99 (68.3)	64 (82.1)	0.019
Diabetes mellitus (*n*, %)	60 (41.4)	41 (52.6)	0.072
Dyslipidemia (*n*, %)	116 (80.0)	68 (87.2)	0.122
Coronary artery disease (*n*, %)	51 (35.2)	35 (44.9)	0.101
Cerebral vascular disease (*n*, %)	17 (11.7)	9 (11.5)	0.577
Chronic kidney disease (*n*, %)	48 (34.3)	37 (54.4)	0.005
Anemia (*n*, %)	31 (22.5)	23 (33.3)	0.067
**Medications**			
β blockers (*n*, %)	126 (86.9)	68 (87.2)	0.565
ACEIs/ARBs (*n*, %)	117 (80.7)	67 (85.9)	0.216
Loop diuretics (*n*, %)	84 (57.9)	49 (62.8)	0.286
Inotropic agents (*n*, %)	16 (11.0)	12 (15.4)	0.233
Calcium blockers (*n*, %)	40 (27.6)	28 (35.9)	0.129
Antiplatelet agents (*n*, %)	71 (49.0)	31 (60.3)	0.070
Anticoagulants (*n*, %)	68 (46.9)	35 (44.9)	0.441
**Laboratory data**			
BNP (pg/mL)	151.6 (43.8–509.4)	207.4 (72.5–431.3)	0.326
**Echocardiographic data**			
LVEF (%)	45.0 (31.9–60.4)	46.0 (36.9–63.0)	0.250
**Cardiopulmonary exercise testing**			
Peak VO_2_ (mL/kg/min)	17.3 (14.4–21.1)	14.6 (12.8–17.7)	<0.001
VE-VCO_2_ slope	30.8 (26.7–34.0)	32.7 (29.1–39.8)	<0.001

*CAVI, cardio-ankle vascular index; NYHA, New York Heart Association; EF, ejection fraction; ACEI, angiotensin converting enzyme inhibitor; ARB, angiotensin receptor blocker; BNP, brain natriuretic peptide; LVEF, left ventricular ejection fraction; VO_2_, oxygen uptake; VE-VCO_2_, ventilatory equivalent vs. carbon dioxide output*.

ROC analysis demonstrated that a CAVI cut-off value of 8.9 predicted impaired exercise capacity (Figure 2; area under the curve 0.67, 95% confidence interval, 0.52–0.69, *P* < 0.05). In the multivariate logistic regression analysis for predicting impaired peak VO_2_ (Table 2), high CAVI was found to be an independent factor (odds ratio 2.343, 95% confidence interval 1.021–5.380, *P* = 0.045).

**Table 2:** Logistic regression analysis for predicting impaired peak VO_2_.

	**Univariate**		**Multivariate**	
	**OR (95% CI)**	***P* value**	**OR (95% CI)**	***P* value**
High CAVI (≥ 8.9)	2.697 (1.481–4.911)	0.001	2.343 (1.021–5.380)	0.045
Age	1.037 (1.012–1.063)	0.003	1.009 (0.977–1.043)	0.584
Male sex	0.224 (0.113–0.446)	<0.001	0.120 (0.049–0.292)	<0.001
Body mass index	1.004 (0.942–1.071)	0.899		
Systolic BP	1.001 (0.991–1.011)	0.864		
Diastolic BP	1.005 (0.992–1.018)	0.469		
Heart rate	1.009 (0.995–1.023)	0.210		
Hypertension	2.477 (1.166–5.261)	0.018	2.245 (0.850–5.926)	0.103
Diabetes mellitus	1.194 (0.668–2.137)	0.550		
Dyslipidemia	1.030 (0.478–2.217)	0.940		
Coronary artery disease	1.623 (0.901–2.925)	0.107		
Cerebral vascular disease	0.719 (0.275–1.882)	0.502		
Chronic kidney disease	3.043 (1.627–5.692)	<0.001	2.137 (0.973–4.694)	0.059
Anemia	2.697 (1.400–5.198)	0.003	1.795 (0.797–4.046)	0.158
log BNP	1.993 (1.162–3.419)	0.012	1.913 (0.989–3.700)	0.054
LVEF	0.999 (0.979–1.020)	0.953		

*OR, odds ratio; CI, confidence interval; CAVI, cardio-ankle vascular index; BP, blood pressure; BNP, brain natriuretic peptide; LVEF, left ventricular ejection fraction*.

**Figure 3:**
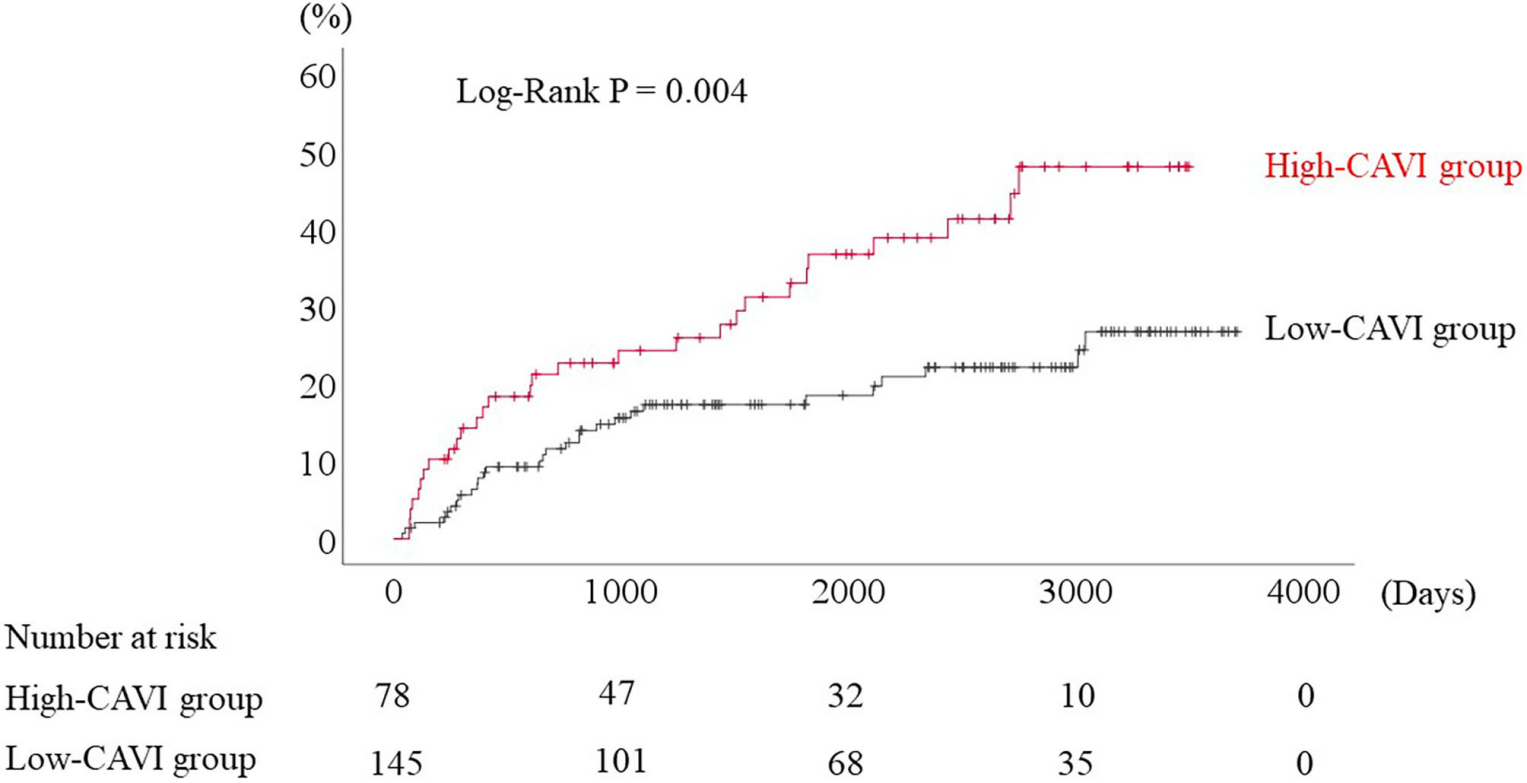
Accumulated cardiac event rates stratified by CAVI.

**Figure 4:**
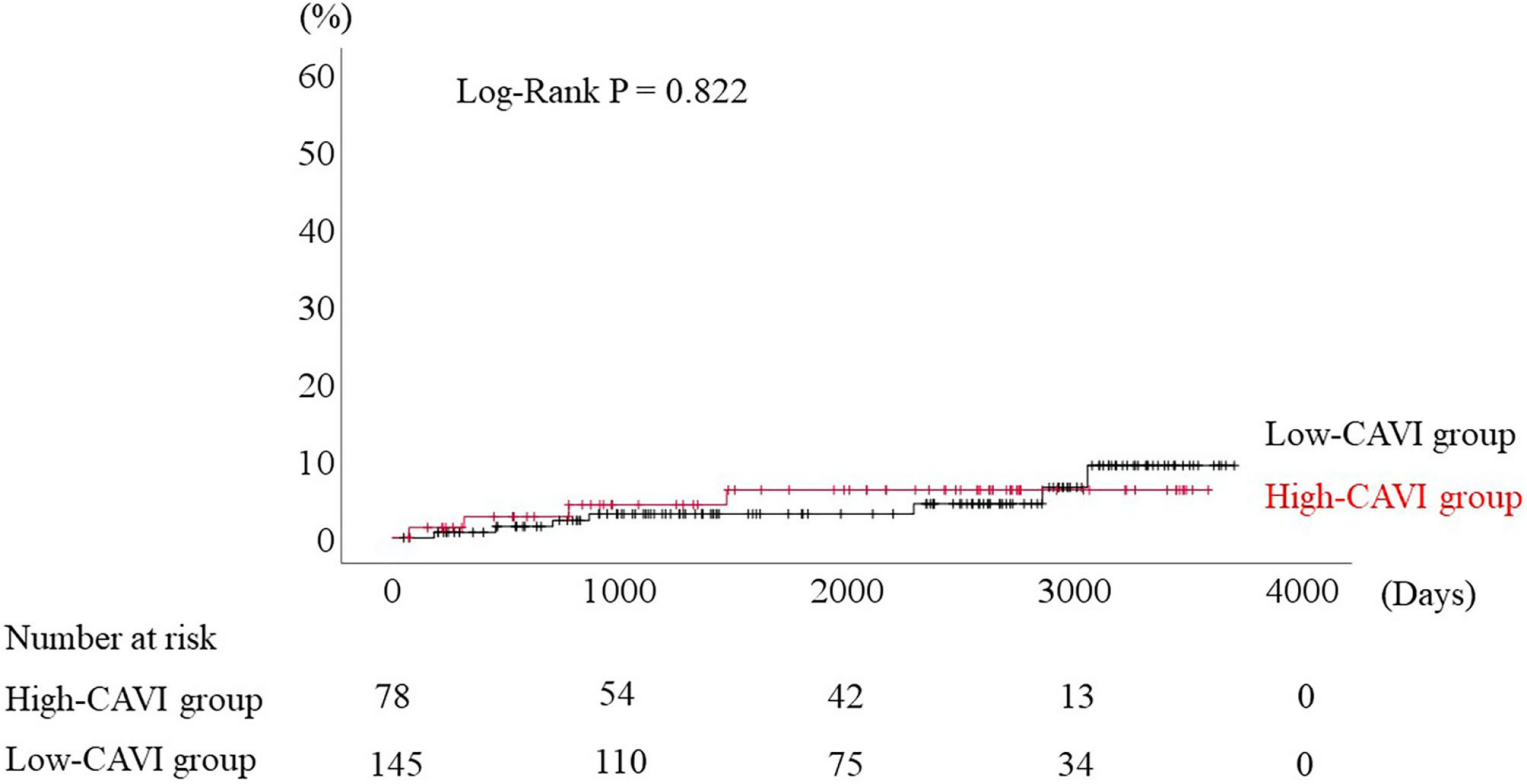
Accumulated all-cause mortality stratified by CAVI.

**Figure 5:**
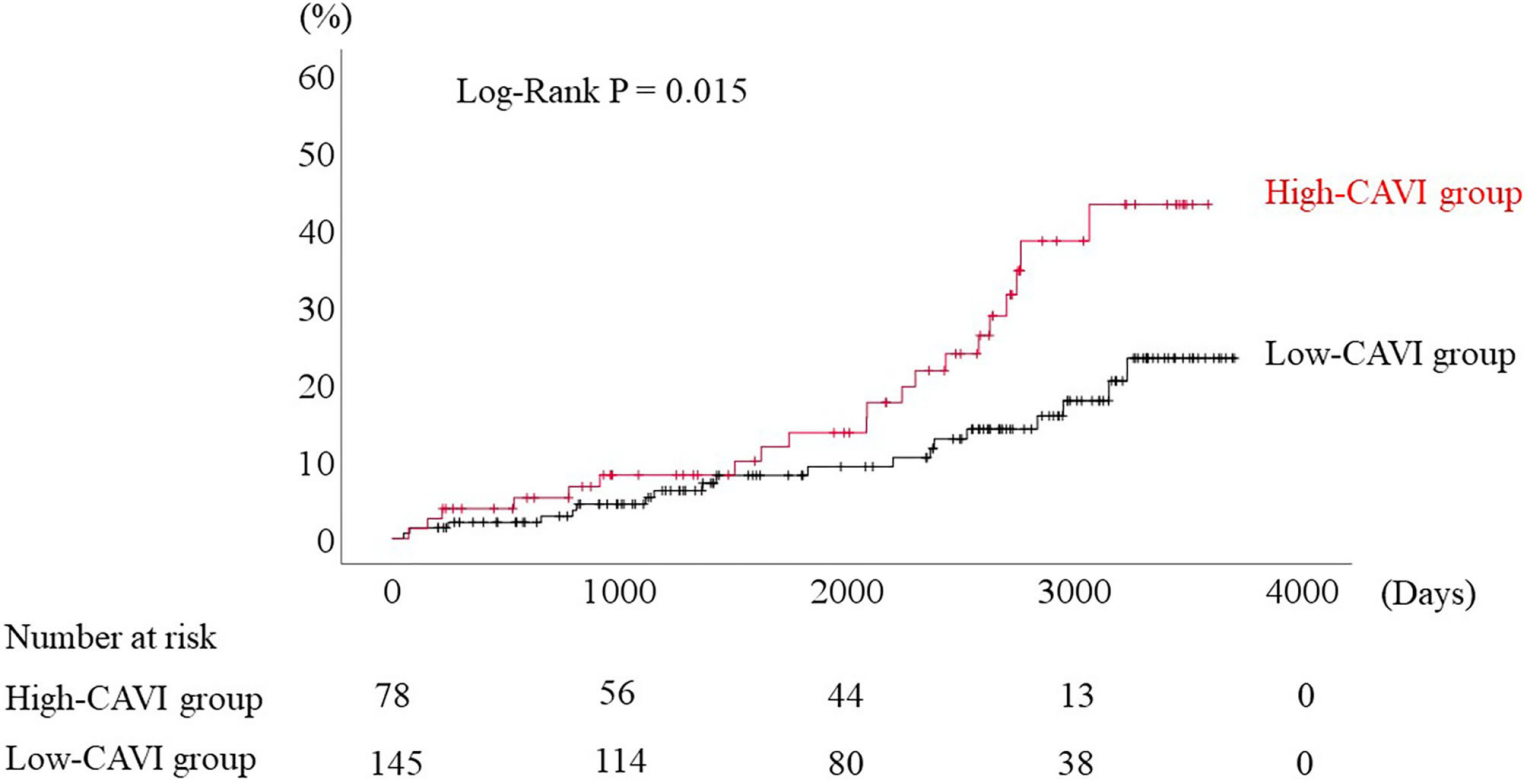
Accumulated ischemic coronary event rates stratified by CAVI.

During the post-discharge follow-up period (median 1,623 days), 58 cardiac events including 53 worsening HF and 5 cardiac deaths, 11 ischemic coronary events and 39 all-cause deaths occurred. The Kaplan-Meier analysis showed that cardiac event rates and all-cause mortality were higher in the high-CAVI group than in the low-CAVI group (Figure 3; cardiac event rates, log-rank *P* = 0.004; Figure 4; all-cause mortality, log-rank *P* = 0.015), however ischemic coronary events did not differ between the high-CAVI and the low-CAVI group (Figure 5; log-rank *P* = 0.822). In the multivariate Cox proportional hazard analysis (Table 3), we considered possible confounding factors, which differed between the groups (i.e., age, sex, body mass index, hypertension, and chronic kidney disease), and high CAVI was found to be an independent predictor of cardiac events (hazard ratio 1.845, 95% confidence interval 1.044–3.261, *P* = 0.035). In contrast, high CAVI did not fully predict ischemic coronary events and all-cause mortality in the multivariate Cox proportional hazard analysis (Tables 4, 5). Furthermore, in the subgroup analysis for predicting cardiac events (Table 6), there was no significant interactions between prognostic impact of CAVI and both sex (*P* = 0.704), age (*P* = 0.291), and LVEF (*P* = 0.279).

**Table 3:** Cox proportional hazard model for cardiac events.

	**Univariate**		**Multivariate**	
	**HR (95% CI)**	***P* value**	**HR (95% CI)**	***P* value**
High CAVI (≥ 8.9)	2.090 (1.248–3.500)	0.005	2.090 (1.248–3.500)	0.005
Age (older vs. younger)	1.520 (0.894–2.585)	0.122		
Male sex	0.771 (0.423–1.408)	0.397		
Body mass index	0.994 (0.933–1.059)	0.858		
Hypertension	1.195 (0.644–2.217)	0.572		
Chronic kidney disease	1.600 (0.933–2.744)	0.088		

*HR, hazard ratio; CI, confidence interval; CAVI, cardio-ankle vascular index*.

**Table 4:** Cox proportional hazard model for ischemic coronary events.

	**Univariate**		**Multivariate**	
	**HR (95% CI)**	***P* value**	**HR (95% CI)**	***P* value**
High CAVI (≥ 8.9)	1.152 (0.336–3.945)	0.822		
Age	1.061 (1.000–1.126)	0.049		
Male sex	0.600 (0.159–2.266)	0.451		
Body mass index	1.065 (0.948–1.196)	0.290		
Hypertension	31.818 (0.095–10611.11)	0.243		
Chronic kidney disease	1.134 (0.318–4.046)	0.846		

*HR, hazard ratio; CI, confidence interval; CAVI, cardio-ankle vascular index*.

**Table 5:** Cox proportional hazard model for all-cause mortality.

	**Univariate**		**Multivariate**	
	**HR (95% CI)**	***P* value**	**HR (95% CI)**	***P* value**
High CAVI (≥ 8.9)	2.145 (1.142–4.027)	0.018	1.802 (0.912–3.561)	0.090
Age	1.027 (1.000–1.055)	0.051	1.018 (0.989–1.048)	0.227
Male sex	1.140 (0.503–2.583)	0.754		
Body mass index	0.921 (0.840–1.010)	0.081		
Hypertension	1.377 (0.607–3.125)	0.445		
Chronic kidney disease	1.612 (0.843–3.081)	0.149		

*HR, hazard ratio; CI, confidence interval; CAVI, cardio-ankle vascular index*.

**Table 6:** Cox proportional hazard model for cardiac events: the impact of high CAVI (Sub-group analysis).

**Factor**	**Subgroup**	***n***	**HR (95% CI)**	***P* value**	**Interaction *P* value**
**Total**			2.090 (1.248–3.500)	0.005	
Sex	Male	178	2.057 (1.138–3.719)	0.017	0.704
	Female	45	2.491 (0.854–7.268)	0.095	
Age	Older (≥ median 61 years)	116	1.802 (0.905–3.586)	0.094	0.291
	Younger (< median 60 years)	107	2.908 (1.133–7.466)	0.026	
LVEF	Reduced and mid-range EF	105	1.934 (1.036–3.608)	0.038	0.279
	Preserved EF	84	3.572 (1.196–10.672)	0.023	

*HR, hazard ratio; CI, confidence interval; CAVI, cardio-ankle vascular index; LVEF, left ventricular ejection fraction; EF, ejection fraction*.

## Discussion

The present study, to the best of our knowledge, was the first to report that (A) high CAVI (≥ 8.9) was independently associated with impaired peak VO_2_ (< 14 mL/kg/min), and (B) high CAVI independently predicted the cardiac events in patients with HF.

There was weak association between CAVI and exercise capacity in the present study. Concordant with our data, it has recently been reported that CAVI was associated with 6-min walk test, and indicated that arterial stiffness may relate to partly exercise capacity (17). Regarding arterial stiffness and impaired exercise capacity in HF patients, abnormal ventricular-arterial coupling may be caused by vascular dysfunction in HF (4). Because of arterial stiffness and an impaired peripheral vasodilatory response to exercise, the timing and amplitude of the reflected pulse wave are changed, and as a result the pulsatile load arriving at the heart during late systole increases (4, 18). After that, the myocardial workload during exercise increases and contributes to functional exercise intolerance (4). Arterial stiffening and abnormal vasorelaxation during exercise elevate filling pressure and impair cardiac output reserve in HF patients, and contribute to exercise intolerance (4, 5, 19). Vascular dysfunction also decreases the O_2_ delivery to the skeletal muscle at the start of exercise, and the skeletal muscle uses anaerobic energy (4, 20). The decrease of the finite energy sources needed to maintain exercise at latter exercise stages contributes to exercise intolerance (4, 20). In addition, CAVI was reportedly to be an independent risk factor for frailty (21), which is associated with adverse outcome in HF patients (22, 23). Arterial hemodynamic dysfunction may have a predictive effect on reduction in muscle mass, and the reduction results in a decrease in body mass, grip strength, and walking speed (21). Muscle blood flow decreases were partly related to the degree of atherosclerosis (24). Therefore, atherosclerosis and arterial stiffness were risk factors for frailty (21, 25).

Especially, in patients with HFpEF, arterial stiffness is increased and is correlated with decreased exercise capacity (5, 26–29). Arterial stiffening and impaired arterial vasodilator reserve with exercise are important in the pathophysiology of HFpEF that is independent of hypertension and mean blood pressure alone (5). A reduction in pulsatile arterial afterload improves functional capacity measured by the 6-min walk test (4, 30). The impairment oxygen delivery and extraction in tissue is considered an important determinant of exercise tolerance (4, 31, 32).

In the present study, CAVI was an independent predictor of impaired peak VO_2_, after adjustment for important factors including age, blood pressure and LVEF. Thus, CAVI may be a useful marker for impaired exercise capacity, especially in HF patients who have difficulty undergoing CPX testing and other exercise tests.

There are stronger relationships between arterial stiffness and HF, because decreases in arterial wall compliance increase cardiac afterload and exacerbate HF (8). Meguro et al. (33) reported that the high brachial-ankle pulse wave velocity (BaPWV) group had a lower event-free survival rate than the low BaPWV group, so elevated arterial stiffness is a risk factor for rehospitalization or cardiac death of HF patients. On the other hand, PWV has a weak point; it is known to depend on blood pressure at the time of measurement, whereas CAVI is independent of blood pressure (6). Consistent with our results that the cut-off value of CAVI was 8.9, a recent review of vascular function has suggested that CAVI ≥ 9.0 is a marker of vascular failure (9). Additionally, it has been reported that CAVI ≥ 9.0 predicted higher cardiovascular events in diabetic patients (34). On the other hand, the associations between changes of CAVI and prognosis have not yet been examined (35). A prospective, large-scale, and longitudinal study with repeated measurement of CAVI in high cardiovascular risk patients, the Coupling registry, has been under way (35). The study may provide useful information on the significance of both baseline CAVI and changes in CAVI over time as indicators of cardiovascular prognosis (35).

Our study has several strengths. For example, to the best of our knowledge, the present study is the first to show associations between increased CAVI and impaired exercise as well as adverse prognosis in HF patients, taking into consideration a multifaceted background and exercise capacity within a given population. Second, we were able to follow up on all patients.

The present study has several limitations. First, since CAVI measurement is inappropriate for patients with concurrent peripheral artery disease and atrial fibrillation, which are sometimes complicated with HF, CAVI is not necessarily indicated for all HF patients. Second, the results of the current study may not represent the general population, as this was a prospective cohort study of a single center with a relatively small number of patients. We considered several confounding factors and performed multivariate Cox proportional hazard analysis, but we cannot exclude all residual confounding factors, and we might not completely adjust for the effects of the differences in the backgrounds between the groups. Third, in the present study we considered the variables during hospitalization for decompensated HF, but we did not analyze the changes in medical parameters (e.g., CAVI) throughout the clinical course and post-discharge treatment. Fourth, although we encouraged CAVI and CPX in hospitalized patients, attending physicians could not perform these measurements in all patients for various reasons (e.g., patient refusal, medical reasons, timing of hospital discharge). Thus, potential selection bias in these measurements possibly existed. Fifth, the present study was a cross-sectional and prospective observational study, therefore we could not fully explain the causal relationships and mechanisms of increased CAVI on impaired exercise capacity and worse prognosis. Therefore, the present results should be considered preliminary, and further studies analyzing larger population are required.

## Conclusion

High CAVI is independently associated with impaired exercise capacity, and leads to a high cardiac event rate in HF patients.

## Data Availability Statement

The raw data supporting the conclusions of this article will be made available by the authors, without undue reservation.

## Ethics Statement

The studies involving human participants were reviewed and approved by The ethical committee of Fukushima Medical University. The patients/participants provided their written informed consent to participate in this study.

## Author Contributions

KW and YS: conceptualization, methodology, formal analysis, investigation, writing–original draft, and visualization. AY: conceptualization, methodology, formal analysis, investigation, resources, data curation, writing–original draft, visualization, supervision, project administration, and funding acquisition. YH, FA, YI, YK, TY, TM, TK, MO, and AK: conceptualization, methodology, formal analysis, investigation, and, writing–review and editing. YT: conceptualization, methodology, formal analysis, investigation, writing–original draft, supervision, and project administration. All authors contributed to the article and approved the submitted version.

## Conflict of Interest

AY and TM belong to the Department of Advanced Cardiac Therapeutics, which is supported by Fukuda-Denshi CO, Ltd. TY and KS belong to the Department of Pulmonary Hypertension, which is supported by ACTELION PHARMA Co, Ltd. These companies are not associated with the contents of the current study. The remaining authors declare that the research was conducted in the absence of any commercial or financial relationships that could be construed as a potential conflict of interest.
