# Supplementary material for: Cardio-Ankle Vascular Index Reflects Impaired Exercise Capacity and Predicts Adverse Prognosis in Patients With Heart Failure
Source: Front Cardiovasc Med. 2021 Mar 29;8:631807. doi: 10.3389/fcvm.2021.631807 (PMC8044779; doi:10.3389/fcvm.2021.631807)
Supplement: Supplementary file 1 [file Data_Sheet_1.docx]

**Supplementary Table 1. Baseline patient characteristics based on age (n = 223)**

|  | **Younger age**  **(age < 61 , n = 107)** | **Older age**  **(age ≥ 61, n = 116)** | **P value** |
| --- | --- | --- | --- |
| **CAVI** | 7.25 (6.43–8.05) | 9.04 (7.87–9.70) | <0.001 |
| **High CAVI** | 15 (14.0) | 63 (54.3) | <0.001 |
| **Demographic data** |  |  |  |
| Age (years old) | 50.0 (41.5–57.0) | 70.0 (65.0–75.0) | <0.001 |
| Male sex (n, %) | 86 (80.4) | 92 (79.3) | 0.488 |
| Body mass index (kg/m^2^) | 25.4 (22.0–28.9) | 23.2 (21.5–25.3) | 0.001 |
| Systolic blood pressure (mmHg) | 124.0 (109.5–146.0) | 127.0 (109.5–144.5) | 0.780 |
| Diastolic blood pressure (mmHg) | 79.0 (64.0–91.5) | 68.0 (60.0–80.0) | 0.005 |
| Heart rate (/min) | 85.0 (68.0–97.5) | 72.0 (61.0–88.0) | 0.002 |
| NYHA functional class Ⅲ or Ⅳ (n, %) | 1 (0.9) | 1 (0.9) | 0.731 |
| Ischemic etiology (n, %) | 28 (30.8) | 42 (42.0) | 0.072 |
| Reduced/mid-range/preserved　EF (n, %) | 37 (39.8)/ 20 (21.5)/ 36 (38.7) | 30 (31.3)/ 18 (18.8)/ 48 (50.0) | 0.286 |
| **Co-morbidities** |  |  |  |
| Hypertension (n, %) | 71 (66.4) | 92 (79.3) | 0.021 |
| Diabetes mellitus (n, %) | 51 (47.7) | 50 (43.1) | 0.494 |
| Dyslipidemia (n, %) | 85 (79.4) | 99 (85.3) | 0.163 |
| Coronary artery disease (n, %) | 38 (35.5) | 48 (41.4) | 0.223 |
| Cerebral vascular disease (n, %) | 7 (6.5) | 19 (16.4) | 0.018 |
| Chronic kidney disease (n, %) | 33 (33.0) | 52 (48.1) | 0.019 |
| Anemia (n, %) | 17 (17.0) | 37 (34.6) | 0.003 |
| **Medications** |  |  |  |
| β blockers (n, %) | 91 (85.0) | 103 (88.8) | 0.264 |
| ACEIs/ARBs (n, %) | 86 (80.4) | 98 (84.5) | 0.264 |
| Loop diuretics (n, %) | 62 (57.9) | 71 (61.2) | 0.620 |
| Inotropic agents (n, %) | 12 (11.2) | 16 (13.8) | 0.354 |
| Calcium blockers (n, %) | 27 (25.2) | 41 (35.3) | 0.067 |
| Antiplatelet agents (n, %) | 49 (45.8) | 69 (59.5) | 0.041 |
| Anticoagulants (n, %) | 50 (46.7) | 53 (45.7) | 0.876 |
| **Laboratory data** |  |  |  |
| BNP (pg/mL) | 139.5 (40.5–548.0) | 177.2 (67.3–431.4) | 0.382 |
| **Echocardiographic data** |  |  |  |
| LVEF (%) | 44.9 (33.1–58.0) | 49.8 (35.6–61.0) | 0.198 |
| **Cardiopulmonary exercise testing** |  |  |  |
| Peak VO_2_ (mL/kg/min) | 17.2 (14.3–21.4) | 15.4 (12.8–18.5) | 0.001 |
| VE-VCO_2_ slope | 30.3 (26.6–33.0) | 33.1 (29.3–38.3) | <0.001 |

CAVI, cardio-ankle vascular index; NYHA, New York Heart Association; EF, ejection fraction; ACEI, angiotensin converting enzyme inhibitor; ARB, angiotensin receptor blocker; BNP, brain natriuretic peptide; LVEF, left ventricular ejection fraction; VO_2_, oxygen uptake: VE-VCO_2_, ventilatory equivalent versus carbon dioxide output.

**Supplementary Table 2. Baseline patient characteristics: patients with older age (n=116)**

|  | **Low CAVI**  **(CAVI < 8.9, n = 53)** | **High CAVI**  **(CAVI ≥ 8.9, n = 63)** | **P value** |
| --- | --- | --- | --- |
| **CAVI** | 7.78 (7.15–8.38) | 9.68 (9.30–10.32) | <0.001 |
| **Demographic data** |  |  |  |
| Male sex (n, %) | 40 (75.5) | 52 (82.5) | 0.240 |
| Body mass index (kg/m^2^) | 23.5 (22.4–25.3) | 22.7 (21.1–25.3) | 0.181 |
| Systolic blood pressure (mmHg) | 127.0 (109.0–138.0) | 130.0 (110.5–150.0) | 0.309 |
| Diastolic blood pressure (mmHg) | 68.0 (60.0–77.0) | 70.0 (60.0–88.0) | 0.200 |
| Heart rate (/min) | 72.0 (61.0–87.0) | 72.0 (62.0–89.0) | 0.684 |
| NYHA functional class Ⅲ or Ⅳ (n, %) | 0 (0.0) | 1 (1.6) | 0.543 |
| Ischemic etiology (n, %) | 20 (42.6) | 22 (41.5) | 0.539 |
| Reduced/mid-range/preserved　EF (n, %) | 15 (33.3)/ 9 (20.0)/ 21 (46.7) | 15 (29.4)/ 9 (17.6)/ 27 (52.9) | 0.828 |
| **Co-morbidities** |  |  |  |
| Hypertension (n, %) | 41 (77.4) | 51 (81.0) | 0.402 |
| Diabetes mellitus (n, %) | 19 (35.8) | 31 (49.2) | 0.104 |
| Dyslipidemia (n, %) | 44 (83.0) | 55 (87.3) | 0.348 |
| Coronary artery disease (n, %) | 23 (43.4) | 25 (39.7) | 0.414 |
| Cerebral vascular disease (n, %) | 10 (18.9) | 9 (14.3) | 0.339 |
| Chronic kidney disease (n, %) | 21 (39.6) | 31 (56.4) | 0.061 |
| Anemia (n, %) | 18 (35.3) | 19 (33.9) | 0.522 |
| **Medications** |  |  |  |
| β blockers (n, %) | 48 (90.6) | 55 (87.3) | 0.401 |
| ACEIs/ARBs (n, %) | 43 (81.1) | 55 (87.3) | 0.255 |
| Loop diuretics (n, %) | 31 (58.5) | 40 (63.5) | 0.359 |
| Inotropic agents (n, %) | 6 (11.3) | 10 (15.9) | 0.333 |
| Calcium blockers (n, %) | 17 (32.1) | 24 (38.1) | 0.316 |
| Antiplatelet agents (n, %) | 32 (60.4) | 37 (58.7) | 0.504 |
| Anticoagulants (n, %) | 26 (49.1) | 27 (42.9) | 0.315 |
| **Laboratory data** |  |  |  |
| BNP (pg/mL) | 158.8 (46.5–431.5) | 234.2 (94.8–431.3) | 0.163 |
| **Echocardiographic data** |  |  |  |
| LVEF (%) | 45.7 (34.1–61.3) | 50.3 (38.1–60.8) | 0.680 |
| **Cardiopulmonary exercise testing** |  |  |  |
| Peak VO_2_ (mL/kg/min) | 15.9 (13.9–19.4) | 14.6 (12.6–17.7) | 0.110 |
| VE-VCO_2_ slope | 32.9 (28.9–36.3) | 33.2 (30.0–39.9) | 0.121 |

CAVI, cardio-ankle vascular index; NYHA, New York Heart Association; EF, ejection fraction; ACEI, angiotensin converting enzyme inhibitor; ARB, angiotensin receptor blocker; BNP, brain natriuretic peptide; LVEF, left ventricular ejection fraction; VO_2_, oxygen uptake: VE-VCO_2_, ventilatory equivalent versus carbon dioxide output.

**Supplementary Table 3. Baseline patient characteristics: patients with younger age (n = 107)**

|  | **Low CAVI**  **(CAVI < 8.9, n = 92)** | **High CAVI**  **(CAVI ≥ 8.9, n = 15)** | **P value** |
| --- | --- | --- | --- |
| **CAVI** | 7.11 (6.15–7.73) | 9.56 (9.49–9.79) | <0.001 |
| **Demographic data** |  |  |  |
| Male sex (n, %) | 72 (78.3) | 14 (93.3) | 0.155 |
| Body mass index (kg/m^2^) | 25.4 (22.0–29.0) | 24.6 (22.9–27.2) | 0.650 |
| Systolic blood pressure (mmHg) | 122.0 (107.5–144.0) | 134.0 (119.0–152.0) | 0.114 |
| Diastolic blood pressure (mmHg) | 75.5 (62.0–91.0) | 87.0 (79.0–104.0) | 0.069 |
| Heart rate (/min) | 85.0 (68.5–100.5) | 79.0 (66.5–88.0) | 0.260 |
| NYHA functional class Ⅲ or Ⅳ (n, %) | 1 (1.1) | 0 (0.0) | 0.860 |
| Ischemic etiology (n, %) | 21 (26.6) | 7 (58.3) | 0.033 |
| Reduced/mid-range/preserved　EF (n, %) | 33 (41.3)/ 15 (18.8)/ 32 (40.0) | 4 (30.8)/ 5 (38.5)/ 4 (30.8) | 0.276 |
| **Co-morbidities** |  |  |  |
| Hypertension (n, %) | 58 (63.0) | 13 (86.7) | 0.061 |
| Diabetes mellitus (n, %) | 41 (44.6) | 10 (66.7) | 0.095 |
| Dyslipidemia (n, %) | 72 (78.3) | 13 (86.7) | 0.361 |
| Coronary artery disease (n, %) | 21 (26.6) | 7 (58.3) | 0.033 |
| Cerebral vascular disease (n, %) | 7 (7.6) | 0 (0.0) | 0.336 |
| Chronic kidney disease (n, %) | 27 (31.0) | 6 (46.2) | 0.219 |
| Anemia (n, %) | 13 (14.9) | 4 (30.8) | 0.153 |
| **Medications** |  |  |  |
| β blockers (n, %) | 78 (84.8) | 13 (86.7) | 0.604 |
| ACEIs/ARBs (n, %) | 74 (80.4) | 12 (80.0) | 0.602 |
| Loop diuretics (n, %) | 53 (57.6) | 9 (60.0) | 0.547 |
| Inotropic agents (n, %) | 10 (10.9) | 2 (13.3) | 0.528 |
| Calcium blockers (n, %) | 23 (25.0) | 4 (26.7) | 0.557 |
| Antiplatelet agents (n, %) | 39 (42.4) | 10 (66.7) | 0.071 |
| Anticoagulants (n, %) | 42 (45.7) | 8 (53.3) | 0.391 |
| **Laboratory data** |  |  |  |
| BNP (pg/mL) | 144.0 (42.1–548.0) | 93.5 (29.0–404.1) | 0.443 |
| **Echocardiographic data** |  |  |  |
| LVEF (%) | 44.9 (31.6–59.1) | 46.0 (36.9–50.0) | 0.682 |
| **Cardiopulmonary exercise testing** |  |  |  |
| Peak VO_2_ (mL/kg/min) | 18.0 (14.5–22.4) | 14.7 (13.4–16.5) | 0.035 |
| VE-VCO_2_ slope | 30.0 (26.5–33.1) | 30.5 (27.6–32.8) | 0.353 |

CAVI, cardio-ankle vascular index; NYHA, New York Heart Association; EF, ejection fraction; ACEI, angiotensin converting enzyme inhibitor; ARB, angiotensin receptor blocker; BNP, brain natriuretic peptide; LVEF, left ventricular ejection fraction; VO_2_, oxygen uptake: VE-VCO_2_, ventilatory equivalent versus carbon dioxide output.
